# Impact of *MYH6* variants in hypoplastic left heart syndrome

**DOI:** 10.1152/physiolgenomics.00091.2016

**Published:** 2016-10-27

**Authors:** Aoy Tomita-Mitchell, Karl D. Stamm, Donna K. Mahnke, Min-Su Kim, Pip M. Hidestrand, Huan Ling Liang, Mary A. Goetsch, Mats Hidestrand, Pippa Simpson, Andrew N. Pelech, James S. Tweddell, D. Woodrow Benson, John W. Lough, Michael E. Mitchell

**Affiliations:** ^1^Department of Surgery, Division of Cardiovascular Surgery and Children's Research Institute, Medical College of Wisconsin, Milwaukee, Wisconsin;; ^2^Department of Mathematics, Statistics and Computer Science, Marquette University, Milwaukee, Wisconsin;; ^3^Department of Pediatrics, and Children's Research Institute, Medical College of Wisconsin, Milwaukee, Wisconsin;; ^4^Department of Cell Biology, Neurobiology and Anatomy, Medical College of Wisconsin Milwaukee, Wisconsin;; ^5^Department of Cardiothoracic Surgery, the Heart Institute, Cincinnati Children's Hospital, Cincinnati, Ohio;; ^6^Department of Pediatrics, Division of Pediatric Cardiology, Pediatric Heart Center, UC Davis Children's Hospital, Sacramento, California; and; ^7^Department of Pediatric Cardiology, Eastern Maine Medical Center, Bangor, Maine

**Keywords:** hypoplastic left heart syndrome, genetics, transplant-free survival outcome, upregulation of contractility genes, cardiomyocyte-autonomous

## Abstract

Hypoplastic left heart syndrome (HLHS) is a clinically and anatomically severe form of congenital heart disease (CHD). Although prior studies suggest that HLHS has a complex genetic inheritance, its etiology remains largely unknown. The goal of this study was to characterize a risk gene in HLHS and its effect on HLHS etiology and outcome. We performed next-generation sequencing on a multigenerational family with a high prevalence of CHD/HLHS, identifying a rare variant in the α-myosin heavy chain (*MYH6*) gene. A case-control study of 190 unrelated HLHS subjects was then performed and compared with the 1000 Genomes Project. Damaging *MYH6* variants, including novel, missense, in-frame deletion, premature stop, de novo, and compound heterozygous variants, were significantly enriched in HLHS cases (*P* < 1 × 10^−5^). Clinical outcomes analysis showed reduced transplant-free survival in HLHS subjects with damaging *MYH6* variants (*P* < 1 × 10^−2^). Transcriptome and protein expression analyses with cardiac tissue revealed differential expression of cardiac contractility genes, notably upregulation of the β-myosin heavy chain (*MYH7*) gene in subjects with *MYH6* variants (*P* < 1 × 10^−3^). We subsequently used patient-specific induced pluripotent stem cells (iPSCs) to model HLHS in vitro. Early stages of in vitro cardiomyogenesis in iPSCs derived from two unrelated HLHS families mimicked the increased expression of *MYH7* observed in vivo (*P* < 1 × 10^−2^), while revealing defective cardiomyogenic differentiation. Rare, damaging variants in *MYH6* are enriched in HLHS, affect molecular expression of contractility genes, and are predictive of poor outcome. These findings indicate that the etiology of *MYH6*-associated HLHS can be informed using iPSCs and suggest utility in future clinical applications.

hypoplastic left heart syndrome (HLHS) is a clinically and anatomically severe form of congenital heart disease (CHD). HLHS, characterized by hypoplasia of the ascending aorta and left ventricle, was first described by Noonan and Nadas (32). HLHS accounts for as much as 4% of subjects with CHD but is responsible for 15–25% of CHD-related mortality (3). The cause of HLHS is unknown in most cases.

Evidence supporting a genetic basis for HLHS includes observations of familial clustering and high heritability (16) and concurrence with specific chromosomal disorders such as Turner and Jacobsen syndrome. Variants in genes such as *GJA1* (7), *NKX2.5* (10), *NOTCH1* (11), and most recently, myosin heavy chain 6 (*MYH6*) (38) as well as observations of syndromic or rare copy number variants in cardiogenic genes (13, 14, 39, 41) have been associated with HLHS. Increased frequency of left-ventricular outflow tract obstructions (LVOTO) such as bicuspid aortic valve (BAV) and coarctation of the aorta (CoA) have been noted in relatives of HLHS subjects (16, 17, 23, 24, 29). Although these studies indicate an underlying genetic basis, known risk factors currently explain <5% of HLHS etiology (16, 17).

In this study, next-generation sequencing of a multigenerational CHD/HLHS family revealed a novel variant in the *MYH6* gene. Although *MYH6* variants have been previously associated with cardiac phenotypes (1, 4, 6, 12, 33, 38), to better understand their role in HLHS we have employed a multifaceted approach including a case-control association study, transcriptome analysis of patient cardiac tissue, clinical outcomes, and the use of patient-specific induced pluripotent stem cells (iPSCs) to model HLHS disease in vitro. Results reveal that a significant percentage of HLHS patients have rare and damaging *MYH6* variants that impact the expression of other sarcomere genes and are predictive of poor clinical outcomes. Moreover, experiments using patient-derived cardiomyocytes indicate that HLHS may have a cardiomyocyte-autonomous etiology that can be investigated via in vitro modeling with iPSCs.

## METHODS

### Study Participants

HLHS was strictly defined by atresia or stenosis of the aortic and mitral valves and hypoplasia of the left ventricle and ascending aorta, with intact ventricular septum (36). Subjects with complex cardiovascular malformations combined with left ventricle hypoplasia (such as unbalanced atrioventricular septal defects or double-outlet right ventricle) were excluded. Subjects with known genetic syndromes (Trisomy 18, 21, or Turner syndrome) or with extracardiac malformations suggestive of a genetic syndrome were also excluded. See supplementary methods: phenotyping cardiac malformations for additional details.^1^ This study is in accordance with the principles outlined in the Declaration of Helsinki and institutionally approved research (IRB) protocols by the Children's Hospital of Wisconsin (CHW, Milwaukee, WI). Subjects were consented through the CHD Tissue Bank (IRB #CHW 06/229, GC 300) and the Wisconsin Pediatric Cardiac Registry (IRB #CHW 09/91, GC 889), IRB-approved research databases housed at CHW prior to inclusion in the study (15, 39). Both biorepositories provided all DNA samples, as well as cardiac tissue, from patients and family members, with associated clinical outcome variables.

### Multistage Approach to Investigate HLHS

**Fig. 1.**
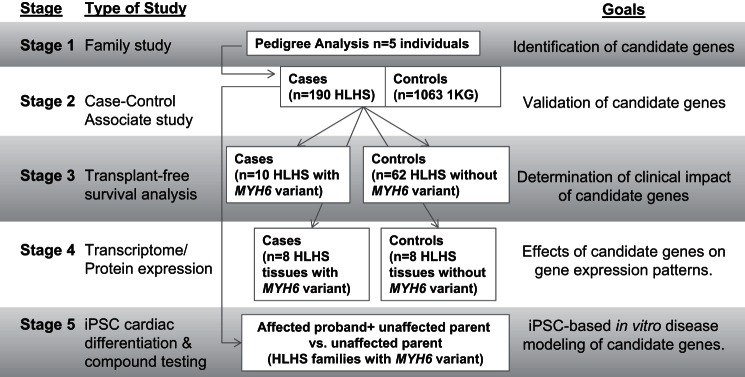
Five-stage study design. *Stage 1* analyzed the pedigree of family F *MYH6*-R443P. *Stage 2* was a case-control association study assessing rare α-myosin heavy chain gene (*MYH6*) variants in 190 unrelated hypoplastic left heart syndrome (HLHS) subjects. *Stage 3* assessed the clinical outcome of *MYH6* variant carriers compared with non-*MYH6* variant carriers. *Stage 4* utilized transcriptome sequencing and Western blotting to compare gene expression in HLHS subjects with and without *MYH6* variants. *Stage 5* employed induced pluripotent stem cells (iPSCs) to model HLHS disease in 2 unrelated families with *MYH6* variants.

Figure 1 depicts the multistage approach used in this study to investigate the role of *MYH6* in HLHS, as follows.

#### Stage 1: multigenerational HLHS family.

**Fig. 2.**
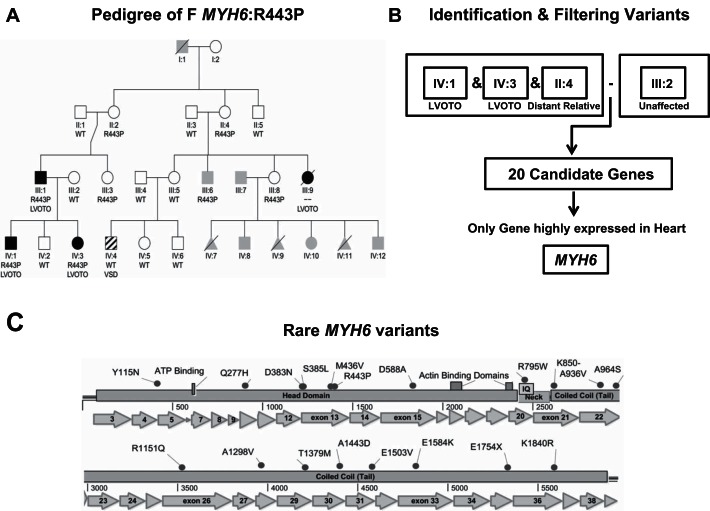
Pedigree of family *MYH6*: R443P, variant filtering scheme and *MYH6* variants in a case-control study. *A*: black squares and circles denote patients with left ventricular outflow tract obstructions (LVOTO; III:1, III:9, IV:1, IV:3). Striping denotes ventricular septal defect, unrelated heart defect to HLHS. Gray-shaded symbols denote subjects with uncertain or no diagnosis of congenital heart disease (CHD). White squares/circles denote patients without CHD. Genotypes denoted R443P indicate heterozygous carriers of an *MYH6* variant. WT, wild type; symbols without a genotype are of unknown genotype. Squares, male; circles, female; triangles, fetal demise. *B*: identification and filtering variants in affected siblings and a distant relative followed by subtracting variants in the unaffected mother identified 20 candidate genes. Among them, *MYH6* was the only candidate highly expressed in the heart. *C*: loci of 19 distinct rare *MYH6* variants discovered in 190 HLHS subjects. *Left* to *right*: loci of variants in the *MYH6* gene encoding the head, neck, and coiled-coil (tail) regions of α-MHC protein. Dots denote variants in HLHS subjects.

We used next-generation sequencing to evaluate a multigenerational family (F *MYH6*-R443P) identified by a proband with HLHS through the CHD Tissue Bank. Other family members had CHDs affecting left-sided heart structures, including a second case of HLHS as shown in the pedigree (Fig. 2). Whole genome sequencing (WGS) was performed on the proband, affected sibling, father, and mother (IV:3, IV:1, III:1, and III:2). Whole exome sequencing (WES) was performed on the paternal great aunt (II:4). For technical processing and variant filtering see supplemental methods: next generation sequencing and supplemental methods: additional stage 1 analysis.

#### Stage 2: case-control association study.

DNA samples were obtained from 190 unrelated HLHS probands. Control genomes were from the 1000 Genomes project (1,063 unrelated individuals of African, Amerindian, Asian, or European descent). (40) Rare, damaging variants (see supplemental methods: next generation sequencing) identified in candidate genes from Stage 1 were individually tested comparing carriers vs. noncarriers in HLHS cases vs. controls (Fisher's exact test).

#### Stage 3: transplant-free survival analysis.

We defined clinical outcomes by comparing transplant-free survival data of HLHS subjects with and without rare, damaging *MYH6* variants. Poor outcome was defined as death or cardiac transplant. Survival was assessed in subjects aged 6 yr or older using Kaplan-Meier curves; a Breslow (generalized Wilcoxon) statistic was used to evaluate significance.

#### Stage 4: mRNA expression/protein expression.

Cardiac tissue discards from HLHS subjects undergoing surgery were snap-frozen in liquid nitrogen and stored at −80°C until RNA or protein isolation. Transcriptome sequencing and Western blot analysis were performed as described in the supplementary appendix. Pairwise analysis was performed, comparing subjects with and without rare, damaging *MYH6* variants matched by age, tissue type, and when possible, by sex and cardiac anatomy (mitral and aortic valve) (Supplemental Table S2). The same matched tissue pairings were also analyzed by Western blot analysis.

#### Stage 5: HLHS modeling with iPSCs.

iPSC lines were generated from dermal fibroblasts donated by two unrelated HLHS probands and their parents (two family trios), denoted as families F *MYH6*-R443P and F *MYH6*-D588A, to the CHD Tissue Bank. Fibroblasts were reprogrammed to pluripotent stem cells using Sendai reprogramming (ReGen Theranostics, Rochester, MN), after which they were returned to CHW (Milwaukee, WI) for experimentation. iPSCs cultured under hypoxic conditions on matrigel in mTeSR1 medium were judged pluripotent from morphological appearance (Supplemental Fig. S3), percentages of cells exhibiting positive Oct4 immunostaining (99–100%), being karyotypically normal (Supplemental Fig. S3), and the ability to differentiate into multiple lineages (definitive endoderm and cardiomyogenic mesoderm) (21). Experimenters were blinded as to the identity of family members from whom each line was generated. Details are described in supplementary materials: ipsc cardiomyocyte differentiation and in ipsc analysis.

## RESULTS

A five-stage approach was employed to elucidate the role of *MYH6* variants in HLHS. In *stage 1*, members of family F *MYH6*-R443P were analyzed with next-generation sequencing identifying a rare variant in the *MYH6* gene. In *stage 2*, a case-control analysis confirmed that rare, damaging *MYH6* variants were highly enriched among 190 unrelated HLHS subjects. *Stage 3* determined the clinical outcome of HLHS subjects with and without rare, damaging *MYH6* variants. *Stage 4* utilized transcriptome sequencing and Western blot analysis to compare gene expression in HLHS subjects with and without rare, damaging *MYH6* variants. *Stage 5* employed iPSCs to model HLHS disease in two unrelated families with different rare, damaging *MYH6* variants.

### Stage 1: Pedigree Analysis of Family F MYH6-R443P

In family F *MYH6*-R443P (Fig. 2*A*) four members with LVOTO were identified. The father (III.1) had CoA, and two children with LVOTO, one of whom is an HLHS proband (IV.3) and the other (IV.1), had double outlet right ventricle with unbalanced atrioventricular canal and hypoplastic left ventricle. A deceased distant relative had a history of HLHS (II:4). One relative had an unrelated heart defect, perimembranous ventricular septal defect (IV:4). Identification and filtering of variants in affected siblings (IV:1 and IV:3) and great aunt (II:4), followed by subtracting variants identified in the unaffected mother (III.2), identified 20 candidate genes (See supplemental methods, next-generation sequencing, and Supplemental Table S1). Among these, *MYH6* was the only gene with a known cardiac association that is highly expressed in the heart (Supplemental Table S1). This established *MYH6* as a candidate gene for this family. A novel R443P mutation in the head/motor domain of *MYH6* was revealed.

### Stage 2: Case-control Association Testing for Rare, Damaging MYH6 Variants in HLHS

To determine *MYH6* association with HLHS, a case-control analysis for rare, damaging *MYH6* variants was performed using a cohort of 190 unrelated HLHS subjects, all of whom had undergone either WES or WGS, with the Thousand Genomes Phase1v3 database serving as a control cohort (See supplemental methods, next-generation sequencing). This revealed the presence of 21 (19 distinct) *MYH6* variants (Table 1, Fig. 2*B*) in 20 (10.5%) HLHS subjects, compared with the presence of rare, damaging *MYH6* variants in only 2.9% of control subjects (*n* = 1,063 unrelated individuals). The null hypothesis that the observed >360% enrichment was due to chance was rejected (Fisher's exact test for difference in proportions *P* < 1 × 10^−5^, Supplemental Table S1). We further validated this finding by performing the same test using a larger database, the Exome Variant Server, National Heart, Lung, and Blood Institute (NHLBI) Gene Ontology Exome Sequencing Project (ESP), Seattle, WA (URL: http://evs.gs.washington.edu/EVS/, date accessed February, 2016) wherein *MYH6* was enriched (*P* < 5 × 10^−3^) (supplemental methods, Supplemental Table S1).

**Table 1.** List of MYH6 variants

Coordinate (b37)	R/A	Domain	Exon (of 39)	Variant Name	Subject	PP2	SIFT	Inherit	Pop Freq
23874837	A/T	head	4	Y115N	R0016	0.999	0	de novo	novel
23872623	C/A	head	10	Q277H	R0735	0.132	0	Mat	0.03%
23870180	C/T	head	13	D383N	07_155	0.986	0	NonPat	novel
23870173	G/A	head	13	S385L	10_121	0.157	0.04	Mat	novel
23870021	T/C	head	13	M436V	10_121	0.996	0.12	NonMat	novel
23869999	C/G	head	13	R443P	07_067	1	0	Pat	novel
23868064	T/G	head	15	D588A	11_099	1	0.26	Mat	0.17%
23865538	G/A	neck	20	R795W	R0023	0.998	0	Mat	0.01%
23863413	CTT/-	tail	21	K850-	07_074	NA	NA	NA	novel
23862995	G/A	tail	22	A936V	R0121	0.796	0.1	Pat	0.02%
23862912	C/A	tail	22	A964S	10_249	0.997	0.07	NA	0.22%
23862912	C/A	tail	22	A964S	09_299	0.997	0.07	NA	0.22%
23859545	C/T	tail	26	R1151Q	07_082	0.937	0.04	NA	novel
23858686	G/A	tail	28	A1298V	09_103	0.65	0.11	NA	0.01%
23858106	G/A	tail	29	T1379M	09_152	0.995	0.06	NA	0.06%
23858106	G/A	tail	29	T1379M	12_093	0.992	0.06	NA	0.06%
23857394	G/T	tail	30	A1443D	11_003	0.885	0.03	NA	novel
23856983	T/A	tail	31	E1503V	12_234	1	0.03	Mat	novel
23855732	C/T	tail	33	E1584K	09_204	0.889	0	Mat	novel
23854153	C/A	tail	35	E1754X	07_026	NA	NA	NA	0.01%
23853696	T/C	tail	36	K1840R	R0300	0.999	0.11	NA	0.02%

Coordinate, start position GRCh37; R, reference allele; A, alternative allele, Subject, identification number; PP2, PolyPhen score; NA, not available; Inherit, inheritance pattern; Mat, maternal; NonMat, nonmaternal; Pat, paternal; NonPat, nonpaternal; Pop Freq, population frequency from ESP 6500 (URL: http://evs.gs.washington.edu/EVS/, accessed November 2015).

All *MYH6* variants in HLHS subjects were confirmed by PCR and Sanger sequencing, or by RNA-Seq analysis, using a different tissue source from the same individual. Among the 19 distinct *MYH6* variants, 10 were novel, one was a three-base pair in-frame deletion, and one was a nonsense mutation. One subject demonstrated paternal inheritance (F *MYH6*-R443P) wherein the father was also affected with CHD, and six subjects demonstrated maternal inheritance patterns (wherein one mother had BAV and five mothers were asymptomatic, although cardiac anatomy was unconfirmed). In addition, one subject exhibited a de novo variant and another demonstrated compound heterozygous inheritance wherein one variant was clearly inherited from the mother (paternal DNA unavailable). Nearly all (19/20) of the HLHS subjects were heterozygous carriers, supporting a previous study suggesting a dominant but incompletely penetrant pattern of inherited atrial septal defects (33). These data confirmed the association (27) of rare, damaging *MYH6* variants with HLHS. Odds ratio was calculated as 4.1 (95% confidence interval 2.3 to 7.4; *P* < 1 × 10^−4^).

### Stage 3: Transplant-free Survival Analysis

**Fig. 3.**
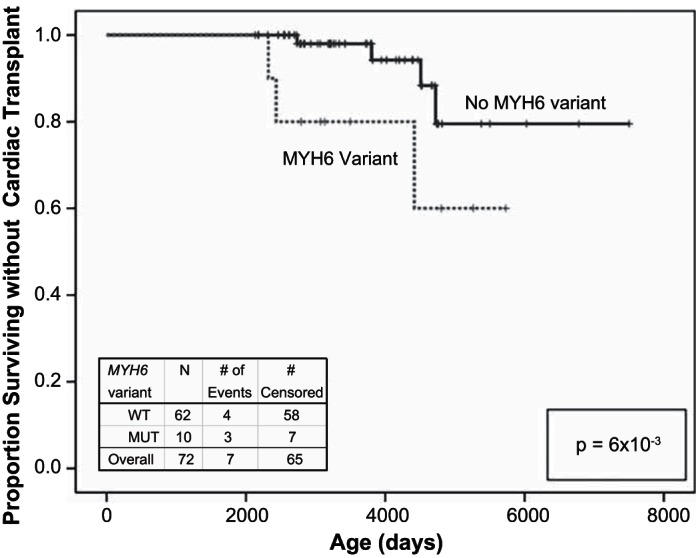
Transplant-free survival analysis. Kaplan-Meier survival curve constructed from 72 HLHS subjects. Among these, 10 subjects had a rare *MYH6* variant (MUT), compared with 62 WT subjects. The Breslow (generalized Wilcoxon) statistic evaluated significance, resulting in rejection of the null hypothesis that these curves are equivalent (*P* = 6 × 10^−3^).

Follow-up data from 72 HLHS subjects (≥6 yr of age) showed that *MYH6* variant carriers (*n* = 10) had significantly lower cardiac transplant-free survival than wild-type subjects (*n* = 62) (*P* < 1 × 10^−2^) (Fig. 3). These data indicate that *MYH6* variants demonstrate a long-term consequence in HLHS pathogenicity (27).

### Stage 4: Increased MYH7 Expression in HLHS Tissues with MYH6 Variants

**Fig. 4.**
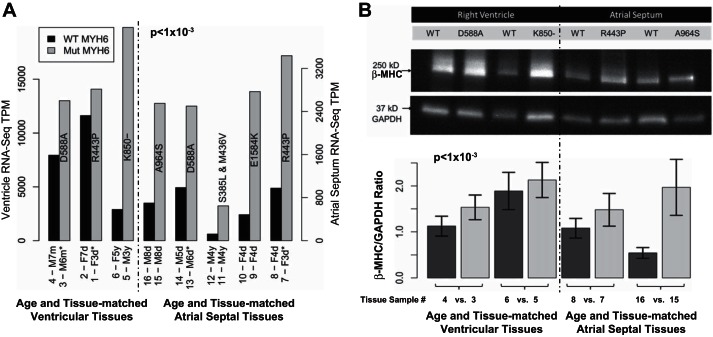
Pairwise analysis of gene expression of *MYH7* in HLHS patients with and without *MYH6* variants. *A*: gray-shaded and black bars denote HLHS patients with and without *MYH6* variants, respectively. The annotation within each gray bar indicates the amino acid change resultant from each *MYH6* mutation. Paired bars on the *left* and *right* side of the figure, respectively, indicate ventricular and atrial septal samples. Subject information is available in Supplemental Table S2. Sample number, sex status (male or female), and age (m, month; d, day; y, year) are denoted in the *x*-axis label. *MYH7* expression is significantly increased (346%) in HLHS patients with an *MYH6* mutation (*P* < 1 × 10^−3^, Supplemental Table S3). TPM, transcripts per million. *B*: Western blot showing increased β-MHC protein in the right ventricle and atrial septum of HLHS patients with an *MYH6* variant. *B, top*: representative Western blot selected from 5 technical replicates of 4 pairs of patient samples; pairings are denoted on the *x*-axis. The entire immunoblot is shown in Supplemental Fig. S2. *B, bottom*: densitometric analysis of β-MHC levels normalized to GAPDH, reflecting the average of 5 replicate determinations performed on the 4 tissue pairs shown in *B, top*. Statistical analysis of the aggregate data representing the 4 pairs was statistically significant *P* < 1 × 10^−3^, generalized linear model/ANOVA, 62%, ± 0.15 SE.

RNA-Seq assessed whether *MYH6* variants affected gene expression in cardiac tissue from HLHS subjects. Transcriptomes were compared in discarded atrial septal tissue from 10 HLHS subjects, half of whom had an *MYH6* variant, as well as from discarded right ventricular tissue of six HLHS subjects, half of whom also had an *MYH6* variant. Tissues were matched pair-wise according to age, and when possible, as well as to sex and aortic and mitral valve anatomy (Fig. 4*A*, Supplemental Table S2). Although no significant change in *MYH6* transcript levels was detected in HLHS tissues containing *MYH6* variants, 22 other genes were differentially expressed (Supplemental Fig. S1, Supplemental Table S3). Among these cardiac troponin T2 (*TNNT2*), myosin heavy chain 7 (*MYH7*), skeletal muscle alpha actin 1 (*ACTA1*), and myosin light chain 2 (*MYL2*) (all which are components of the contractile apparatus), were significantly upregulated (>3- to 12-fold, *P* ≤ 5 × 10^−3^). *MYH7*, the major myosin in human ventricle and the closest paralog of *MYH6*, was increased by almost 350% relative to tissues in HLHS subjects containing wild-type *MYH6* (Fig. 4*A*, Supplemental Table S3; *P* < 1 × 10^−3^). Upregulation of *MYH7* expression from the five pairs of atrial samples was confirmed by quantitative RT-PCR (supplemental methods, *myh7* quantitative rt-pcr). To assess whether increased *MYH7* expression extended to the protein level, levels of β-MHC protein in the same pair-matched atrial septal and right ventricular discards were compared by Western blotting; this revealed that β-MHC was substantially increased in HLHS subjects with *MYH6* variants (generalized linear model/ANOVA, +62%, ± 0.15 SE, *P* < 1 × 10^−3^) (Fig. 4*B*, Supplemental Table S4).

### Stage 5: Increased MYH7 Expression and Defective Differentiation in iPSC-derived Cardiomyocytes from HLHS Subjects with MYH6 Variants

**Fig. 5.**
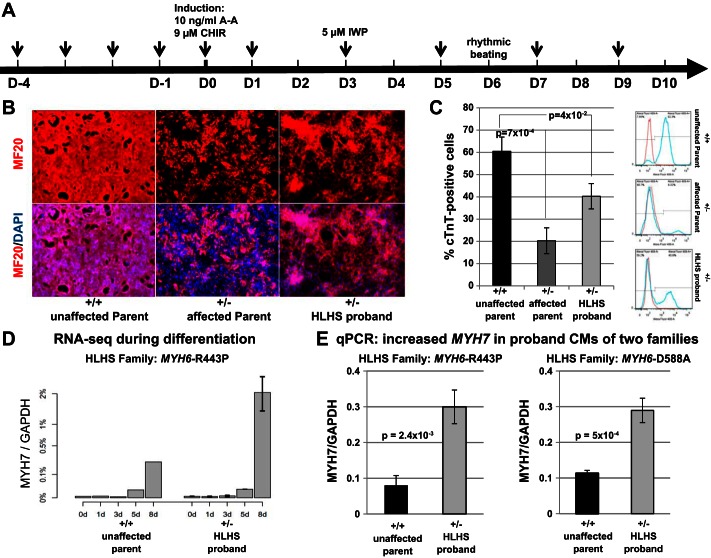
Increased *MYH7* expression in iPSC-derived cardiomyocytes from probands in 2 HLHS families. *A*: scheme for differentiating cardiomyocytes with small molecule Gsk3 inhibitor (CHIR99021) and wnt inhibitor (IWP). *B*: immunostaining of α-MHC at *day 10* showing defective cardiomyogenesis in iPSCs from the HLHS proband and affected parent (carrier father) of family F *MYH6*-R443P. *C*: flow cytometry of cells cultured in parallel with those in *B*, showing decreased percentages of cardiac troponin T (cTnT)-positive cells at *day 10*. Data were compiled from 3 iPSC lines derived from each individual. The *P* values were calculated by Student's *t*-test (2-tailed, equal variance); vertical lines = ± SE. *D*: RNA-Seq showing *MYH7* expression in iPSC-derived cardiomyocytes (CMs) from family F *MYH6*-R443P at differentiation *day 8*. Proband bars represent the average of 2 cell lines; vertical lines = range. Unaffected parent bars represent values from single cell line. *E, left*: quantitative PCR showing increased *MYH7* expression in the HLHS proband of family F *MYH6*-R443P. *E, right*: a similar result from iPSC-derived cardiomyocytes of a separate HLHS family (F *MYH6*-D588A). Bars represent the average of triplicate cultures evaluated in 2 independent iPSC lines (*n* = 6). The *P* values were calculated by Student's *t*-test (2-tailed, equal variance); vertical lines = ± SE. Additional iPSC analysis from family F *MYH6*-D588A is shown in Supplemental Fig. S4.

**Fig. 6.**
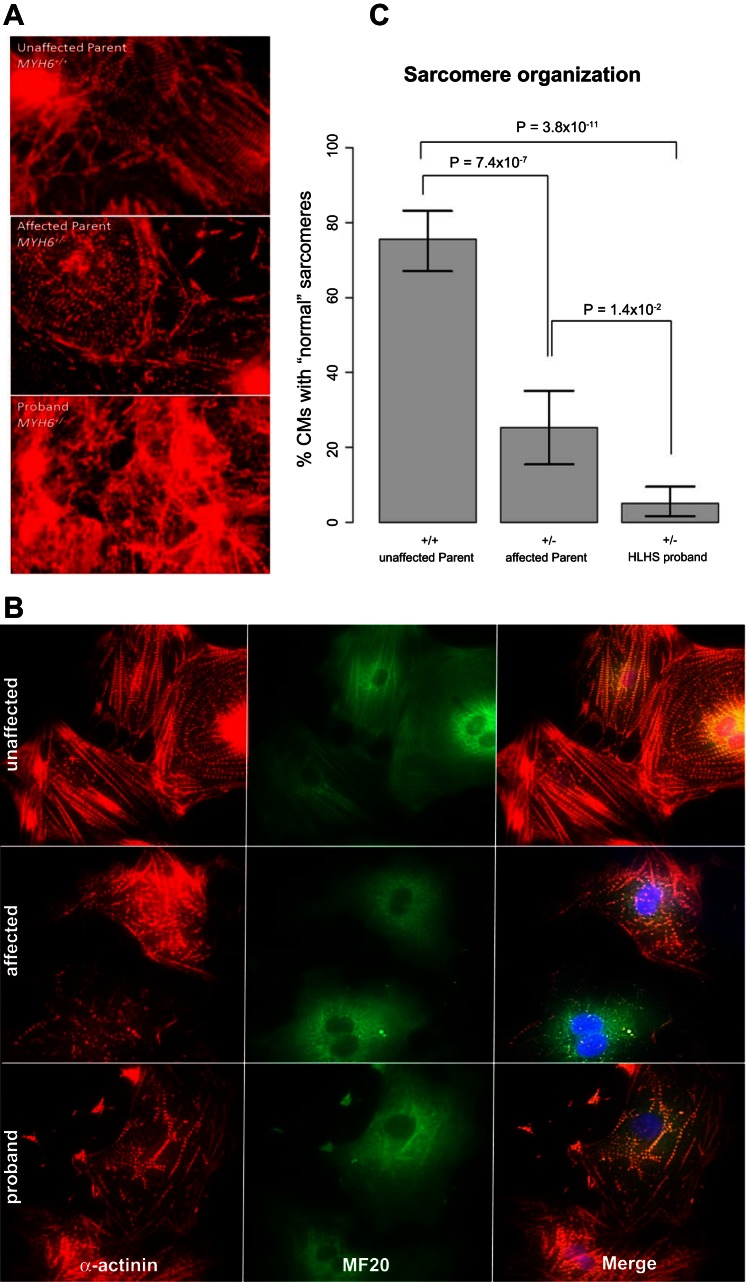
Dysmorphic sarcomeres in CMs derived from iPSCs reprogrammed from the HLHS proband and its affected parent. *A*: α-actinin immunostaining of mass cultured CMs at differentiation *day 65*. *B*: comparative sarcomere organization in individual CMs isolated from the cultures shown in *A* and subcultured at low density. A minimum of 100 isolated CMs representing each individual were judged to contain dysmorphic sarcomere organization if most of the myocyte area displayed blurred staining in which sarcomeric ladders contained punctate or truncated deposits of α-actinin ladders, rather than the relatively crisp and elongated ladders with relatively wide Z-bands that characterize *MYH6*^*+/+*^ cells. *C*: data were compiled from quadruplicate dishes representing each iPSC line (1 line from each parent; 2 lines from the proband) evaluated during a single determination. The *P* values were calculated by Student's *t*-test (2-tailed, equal variance); vertical lines = ± SE.

To determine whether increased *MYH7* expression is phenocopied in cardiomyocytes derived from iPSCs, iPSC lines were generated from dermal fibroblasts of family F *MYH6*-R443P. Following cellular expansion and verification of pluripotency, iPSCs representing the proband and an unaffected parent were induced to undergo cardiomyogenic differentiation as recently described (21) (depicted in Fig. 5*A*), during which cultures of cardiomyogenic cells were removed for RNA-Seq determinations on the differentiation days shown in Fig. 5*D*. As anticipated (21), *MYH7* transcription was first detected on differentiation *day 5*, at which stage similar levels of *MYH7* transcripts were seen in cardiomyocytes representing the proband and its unaffected parent. On *day 8*, although substantial increases in *MYH7* expression were seen in cardiomyocytes from both individuals, the level of expression in proband cardiomyocytes was ∼2.5-fold greater than that observed in myocytes from the unaffected parent. Increased *MYH7* expression in proband cardiomyocytes was confirmed by quantitative PCR performed on two independent iPSC lines from each subject (Fig. 5*E*, *left*). In addition, the efficiency of cardiomyogenic differentiation was significantly reduced in the HLHS proband's cardiomyocytes, as determined by α-MHC immunostaining (Fig. 5*B*) and flow cytometry assessments of percent cTnT-positive cells (Fig. 5*C*). All of these findings were recapitulated in iPSC-derived cardiomyocytes from an unrelated HLHS family that contained a different *MYH6* mutation, which was identified in the CHD Tissue Bank (F *MYH6*-D588A: Fig. 5*E*, *right*, and Supplemental Fig. S4). Finally, we evaluated sarcomere structure in iPSC-derived cardiomyocytes at later stages of differentiation (*days 62–68*), observing that, compared with the unaffected parent, sarcomeres were substantially disorganized in both the affected parent and the proband of family F *MYH6*-R443P (Fig. 6).

## DISCUSSION

Among hypotheses explaining left ventricle hypoplasia in HLHS are that *1*) reduced blood flow due to valvular atresia/stenosis alters ventricular preload with consequent dysmorphology (16) and that *2*) defective expansion and/or differentiation of cardiomyocytes results in dysmorphology and dysfunction (8). While the results reported here can be reconciled with both hypotheses, our observations that iPSC-derived cardiomyocytes from the affected parent and proband of families that carry selected *MYH6* variants undergo poor cardiomyocyte differentiation followed by poor sarcomere organization suggest that HLHS etiology is cardiomyocyte autonomous (Fig. 6). In either event, the data demonstrate that rare *MYH6* variants, which are present in 10.5% of HLHS cases, have pathogenic consequences.

We unexpectedly found that expression of a subset of cardiac contractility genes was increased in the presence of *MYH6* variants. This possibly represents an adaptive mechanism, similar but not identical to that which occurs in the failing heart muscle (35). Most remarkably, the presence of *MYH6* variants is accompanied by strongly increased expression of *MYH7*, in both atrial and ventricular tissues of HLHS subjects (Fig. 4). In addition to confirming the increased expression of *MYH7* in HLHS tissues containing *MYH6* variants (Fig. 4, *A* and *B*), the findings in Fig. 5, *D* and *E*, that increased *MYH7* expression is phenocopied in cardiomyocytes from iPSCs in two separate families, indicate that HLHS etiology can be investigated at the earliest stages of cardiomyogenesis using this in vitro disease model.

Altered contractility has been proposed as an etiologic mechanism in CHD (18). The increase in *MYH7*, which is considered as the “slow twitch isoform,” may adaptively reduce energy requirements in the hypoplastic myocardium because β-MHC (*MYH7*) has relatively low ATPase activity (31). It follows that maximal shortening velocity and peak power would be altered, resulting in hypocontractility. Because *MYH6* is the predominant atrial isoform, it is plausible that atrial hypocontractility due to mutated *MYH6* impedes the flow of fetal blood from the right atrium to the left atrium and through the mitral valve, resulting in limited filling of the left ventricle. This scenario, which is consistent with both the cardiomyocyte-autonomous and flow hypotheses, may explain underdevelopment of the mitral valve, left ventricle, and aortic arch, all of which are hallmarks of HLHS. Indeed, previous studies have shown that mutation of *myh6* in zebrafish and the subsequent disruption of atrial function have a profound effect on ventricular morphogenesis and atrioventricular valve formation (2, 19).

Although examination of the effect of *MYH6* variants on contractile power was beyond the scope of this study, it is noteworthy that variants were observed across all functional domains of α-MHC. In particular, seven mutations were noted in the head/motor domain, including four within an exon 13 “hotspot” that includes R443P (Fig. 3). Alterations in the α-MHC head/motor region have been shown to impact stiffness that results in diastolic dysfunction (20). Also, because the head/motor domain contains binding sites for ATP and actin, variants may induce conformational changes that compromise actin-myosin association. Although only one mutation was found in the neck region, 11 were noted in the coiled-coil/tail region, among which half were located in the domain that interfaces with myosin light chain; in this regard it may be relevant that *MYL2*, which is required for cardiomyocyte differentiation in the murine heart (5), like *MYH7*, also exhibits increased expression in HLHS patients who carry *MYH6* variants (Fig. 4 and Supplemental Table S3). It is also plausible that alterations in the α-MHC tail cause reduced power, as mutations in the tail of homolog β-MHC reportedly distort helicity of the coiled-coil region (42).

Previous studies have found that failing hearts, such as those from cardiomyopathy patients, exhibit decreased levels of *MYH6* mRNA (25, 26, 30), with concomitantly increased expression of *MYH7* (34). It is well known that treatment of cardiomyopathy with β-blockers improves myocardial function and increases survival, an effect that likely results from decreased adrenergic stimulation (9). However, β-blockers also distort the expression of myocardial genes, most remarkably depressing levels of β-MHC concomitant with the restoration of fast-contracting α-MHC fibers (22), resulting in improved cardiac function (25). While acknowledging the complexity of drug-gene interactions, these findings, when superimposed on the results reported here, invite speculation that β-blocker therapy, with or without recently developed cardiac myosin activation therapies (28), may be useful for treating HLHS subjects who carry pathogenic *MYH6* variants. Ongoing work using iPSC-derived cardiomyocytes will determine the responsiveness of myocytes containing pathogenic *MYH6* variants to pharmaceutical agents including β-blockers and myosin activation drugs.

### Limitations

In case-control association analyses, it is desirable to select appropriate control cohorts including those that share ethnic, sex, and age composition. In this study, the publicly available Thousand Genomes Phase 1v3 database was used as a control cohort against whole exomes and genomes of HLHS patients. The controls often had low read-depth and were not imputed; therefore, rare variants may be underrepresented. However, if missed variants exist uniformly across the control genomes, the corresponding decrease in signal-to-noise would not impact our finding that *MYH6* was among the most overrepresented of the 20 candidate genes identified through pedigree analysis. We mitigate genome-wide false positives by only investigating the 20 candidate genes from the pedigree analysis and by replicating enrichment against the larger exome database, NHLBI's ESP.

## GRANTS

This work was supported by National Heart, Lung, and Blood Institute Grant HL-089471 (J. W. Lough) and from the Todd and Karen Wanek Family program for Hypoplastic Left Heart Syndrome (A. Tomita-Mitchell, J. W. Lough, M. E. Mitchell). In addition, this publication is funded in part by the Research and Education Program Fund, a component of the Advancing a Healthier Wisconsin endowment at the Medical College of Wisconsin Grant #5520361 (A. Tomita-Mitchell and J. W. Lough), the Wolfe Family Foundation, the Little Hearts for Life Foundation, the Medical College of Wisconsin's Department of Surgery, and the CRI.

## DISCLOSURES

A. Tomita-Mitchell and M. E. Mitchell are cofounders of Ariosa Diagnostics (San Jose, CA), a prenatal diagnostics biotechnology company that was acquired by Roche in 2015. A. Tomita-Mitchell and M. E. Mitchell are cofounders of TAI Diagnostics (Milwaukee, WI), a biotechnology company involved in transplant diagnostics, and members of its scientific advisory board.

## AUTHOR CONTRIBUTIONS

A.T.-M., K.D.S., M.-S.K., P.M.H., H.L.L., M.H., and J.W.L. analyzed data; A.T.-M., K.D.S., D.K.M., M.-S.K., P.M.S., and J.W.L. interpreted results of experiments; A.T.-M., K.D.S., D.K.M., M.-S.K., P.M.H., P.M.S., and J.W.L. prepared figures; A.T.-M. and M.H. drafted manuscript; A.T.-M., K.D.S., D.K.M., M.-S.K., P.M.H., H.L.L., M.A.G., M.H., A.N.P., J.S.T., D.W.B., and J.W.L. edited and revised manuscript; A.T.-M., K.D.S., D.K.M., M.-S.K., A.N.P., J.S.T., D.W.B., J.W.L., and M.E.M. approved final version of manuscript; K.D.S., D.K.M., M.-S.K., P.M.H., H.L.L., M.A.G., M.H., and J.S.T. performed experiments.

## Supplementary Material

Supplemental Material

## Footnotes

1 The online version of this article contains supplemental material.
