# Supplementary material for: Impact of MYH6 variants in hypoplastic left heart syndrome
Source: Physiol Genomics. 2016 Oct 27;48(12):912–21. doi: 10.1152/physiolgenomics.00091.2016 (PMC5206387; doi:10.1152/physiolgenomics.00091.2016)
Supplement: Supplemental Material [file Supplemental_Material.docx]

**Supplementary Appendix**

**Table of Contents**

Supplemental Methods

- *Phenotyping cardiac malformations*
- *Next generation sequencing*
- *Additional Stage 1 analysis*
- *Variant validation by Sanger sequencing*
- *Cardiac transcriptome sequencing*
- *Cardiac transcriptome data analysis*
- *MYH7 quantitative RT-PCR*
- *Western blotting & densitometry*
- *iPSC karyotyping*
- *iPSC 🡪 cardiomyocyte differentiation*
- *iPSC analysis - immunostaining & flow cytometry & qPCR preparation*

Table S1: Candidate gene list from a family pedigree analysis of F *MYH6*-R443P.

Table S2: Demographic and extended information for the selected 16 RNA samples.

Table S3: Paired test between eight *MYH6*-variant carrying tissue samples and eight wildtype tissue samples in HLHS subjects.

Table S4: Expression level of β-MHC protein assessed by western blot.

Figure S1: Volcano plot of differential gene expression.

Figure S2: Western blot analysis.

Figure S3: Karyotype and colony morphology of iPSCs derived from the HLHS proband and unaffected parent of family *MYH6*-R443P.

Figure S4: iPSC-derived cardiomyocytes from the HLHS proband and affected parent of family F *MYH6*-D588A also exhibit defective cardiomyogenesis (vs. unaffected parent).

**SUPPLEMENTAL METHODS**

*Phenotyping cardiac malformations*

Anatomic cardiac malformations were characterized by phenotyping according to the European Pediatric Cardiac Code (EPCC) and the Society of Thoracic Surgeons (STS)/European Association of Cardio-Thoracic Surgery (EACTS) coding systems. All phenotypes were initially reviewed by a cardiologist. All discrepancies were reconciled by review of source documents including operative notes, echocardiograms, and review by the operative surgeon. Anatomic phenotypes were reported using EPCC terms.([10](#_ENREF_10))

*Next generation sequencing*

Whole Genome Sequencing (WGS) and Whole Exome Sequencing (WES) were performed using a TruSeq library kit with a high depth of coverage (average >30x WGS, 100x WES) on a HiSeq 2000 DNA sequencer (Illumina, San Diego, CA) at the Children’s Hospital of Wisconsin (CHW) Children’s Research Institute (Milwaukee, WI) or the Mayo Clinic (Rochester, MN); WES libraries were generated using the SureSelectXT Human All Exon kit (v4 or v5; Agilent Technologies, Santa Clara, CA) and multiplexed at four exomes per lane at the Children’s Research Institute (Milwaukee, WI). Cluster generation (v3) was carried out using the C-Bot cluster system, and sequencing was accomplished using a HiSeq 2000 sequencer with the 2x100 bp paired-end module (Illumina). Reads were mapped (~96.5%) to the human genome (version GRCh37) using Burrows-Wheeler Aligner.([5](#_ENREF_5)) Variants were identified using GATK([6](#_ENREF_6)) and processed with GEMINI([9](#_ENREF_9)) and Ensembl VEP (v78).([3](#_ENREF_3), [7](#_ENREF_7), [9](#_ENREF_9))

Rare, damaging variants were then identified by considering only variants that satisfied all of the following criteria. Variants could not be intronic, occur within an untranslated region, or be coding-synonymous. In addition, all variants must be novel, or have a minor allele frequency of less than 1% (defined by the highest reported frequency among the NHLBI GO Exome Sequencing Project (ESP) EA and AA subgroups (URL: http://evs.gs.washington.edu/EVS/) [6500 samples, February, 2013]), and the 1000 Genomes Project ASN, AMR, AFR, EUR and SAS subgroups),([12](#_ENREF_12)) such that retained variants were < 1% among any ethnicity. Additional variant requirements were GATK quality score > 66 per subject, base pair locus conservation as indicated by Genomic Evolutionary Rate Profiling (GERP) >1,([2](#_ENREF_2)) and the variant could not lie within a RepeatMasker domain or occur as a known segmental duplication. Finally, the variant was identified as damaging to protein function as defined by PolyPhen2 >0.75 and/or by SIFT<0.30.([1](#_ENREF_1), [8](#_ENREF_8)) To remove technical artifacts, indel calls were maximally 4 bp in length, the site was called confidently among at least half of the cohort, and the indel not found in more than 10% of sequenced subjects.

*Additional Stage 1 analysis*

In Stage 1 of the study, WGS and WES performed on family F *MYH6* R443P were analyzed. This resulted in the identification of 20 candidate genes (**Table S1**) after filtering (two pseudogenes, IGLV5-45 and OR4C4P, were also picked up but not included in the table). This list was further narrowed by focusing on genes that are active in neonatal and pediatric cardiac tissue. To determine high expressing cardiac genes, we used samples described in Stage 4. Briefly, expression levels determined by transcriptome sequencing performed on cardiac tissue discards (N=16) revealed a long-tailed distribution, such that log of expression was approximately normally distributed (mean=0.11, SD=1.03, max=4.81 log10 TPM). Considering this distribution, “highly expressed genes”, i.e. those with expression levels greater than half the maximum observed log10 TPM, corresponded to the 99th percentile among 38,662 transcripts. Based on this result, only *MYH6* was highly expressed among the 20 candidate genes (**Table S1**).

| Transcript | Gene | Variants in Cases | Variants in Controls# | Fisher Exact Test | TPM Atrium | TPM Ventricle |
| --- | --- | --- | --- | --- | --- | --- |
| NM_022060 | *ABHD4* | 1 / 170 | 7/1063 | 6.96 x 10^-1^ | 17.8 | 24.1 |
| NM_001153 | *ANXA4* | 2 / 170 | 6/1063 | 3.04 x 10^-1^ | 23.2 | 12.1 |
| NM_014739 | *BCLAF1* | 1 / 170 | 3/1063 | 4.48 x 10^-1^ | 19.4 | 18.2 |
| NM_001204425 | *BIVM-ERCC5* | 4 / 170 | 18/1063 | 3.60 x 10^-1^ | 7.6 | 7.6 |
| NM_006696 | *BRD8* | 3 / 170 | 17/1063 | 5.35 x 10^-1^ | 25.4 | 22.7 |
| NM_014141 | *CNTNAP2* | 7 / 170 | 22/1063 | 9.22 x 10^-2^ | 0.2 | 0.7 |
| NM_001042517 | *DIAPH3* | 11 / 170 | 20/1063 | 1.73 x 10^-3^ | 3.3 | 0.7 |
| NM_005766 | *FARP1* | 6 / 170 | 32/1063 | 4.29 x 10^-1^ | 52.4 | 25.7 |
| NM_005279 | *GPR1* | 1 / 170 | 11/1063 | 8.33 x 10^-1^ | 1.1 | 0.8 |
| NM_001099650 | *GXYLT1* | 0 / 170 | 3/1063 | 1.00 x 10^0^ | 1.6 | 1.3 |
| NM_002286 | *LAG3* | 4 / 170 | 3/1063 | 8.72 x 10^-3^ | 0.6 | 0.5 |
| NM_005097 | *LGI1* | 0 / 170 | 2/1063 | 1.00 x 10^0^ | 0.8 | 0.2 |
| NM_002471 | *MYH6** | 21 / 190* | 31/1063 | 5.66 x 10^-6^ | 6,190.8 | 978.8 |
| NM_006177 | *NRL* | 5 / 170 | 2/1063 | 7.84 x 10^-4^ | 1.2 | 1.4 |
| NM_002801 | *PSMB10* | 0 / 170 | 0/1063 | 1.00 x 10^0^ | 44.7 | 51.8 |
| NM_004914 | *RAB36* | 3 / 170 | 0/1063 | 2.58 x 10^-3^ | 2.5 | 1.6 |
| NM_152527 | *SLC16A14* | 1 / 170 | 3/1063 | 4.48 x 10^-1^ | 1.5 | 2.8 |
| NM_178498 | *SLC5A12* | 5 / 170 | 7/1063 | 1.65 x 10^-2^ | 4.2 | 0.0 |
| NM_173059 | *ZAN* | 11 / 170 | 18/1063 | 9.11 x 10^-4^ | 0.0 | 0.0 |
| NM_015113 | *ZZEF1* | 13 / 170 | 35/1063 | 9.81 x 10^-3^ | 12.5 | 15.4 |

**Table S1. Candidate gene list from a family pedigree analysis of F *MYH6*-R443P** (columns 1 and 2 denote related transcript and gene name) followed by HLHS case-control comparison (HLHS cases vs 1063 controls with identical filtering in columns 3,4,5) and gene expression levels from the 16 samples of Stage 4 (columns 6 and 7; atrium n=10, ventricle n=6). Case/control analyses cover the coding region of the gene. 170 exomes were available all genes tested; 20 additional WGS were included for *MYH6* therefore the denominator is 190. Fisher Exact Test was applied on filtered variants within HLHS cases compared to filtered variants identified in the control cohort. Fisher Exact Test is the one-tailed p-value for a greater frequency of variants in the HLHS cohort. Seven of the 20 genes were significant (p<1x10^-2^) when compared to the 1KG. Further testing (data not shown) was performed against the larger NHLBI ESP database, which found only two of the 20 candidate genes, *NRL* (p<1x10^-4^) and *MYH6* (p<5x10^-3^), were still significantly enriched at p<1x10^-2^ (Exome Variant Server, NHLBI GO Exome Sequencing Project (ESP), Seattle, WA (URL: http://evs.gs.washington.edu/EVS/) [date accessed (February, 2016)]). The only gene with significant cohort enrichment and high expression in heart was *MYH6*. No genes are ambiguously near the TPM=255 limit of defined “high” expression.

*Variant validation by Sanger sequencing*

Double-stranded DNA generated by PCR amplification was purified using ExoSAP-IT (Affymetrix, Santa Clara, CA), followed by direct sequencing. Sequencing was performed by Retrogen Inc. (San Diego, CA) and analyzed using Sequencher v4.5 (Gene Codes Corporation, Ann Arbor, MI) software.

*Cardiac transcriptome sequencing*

RNA was isolated from cardiac tissue (~10 mg) by enzymatic digestion using an Ambion MELT kit (Invitrogen, New York), which includes an “on-bead” DNase digestion step to remove contaminating genomic DNA. RNA was isolated from differentiating cardiomyocytes *in vitro* using an RNeasy minikit according to the manufacturer’s (Qiagen, Valencia, CA) instructions. Isolated RNA was of high quality as determined by Bioanalyzer 2100 RNA Integrity Number (range: 7-9.5; Agilent Technologies, CA). RNA sequencing libraries were prepared from 500 ng total RNA using the Illumina TruSeq kit (version 2.5). Samples were spiked with external RNA controls (ERCC sequences, Ambion Life Technologies, Grand Island, NY). Unique indices were introduced according to the protocol, in order for sample multiplexing during the sequencing run. Library quantitation was accomplished by qPCR and subsequent sequencing was carried out on an Illumina HiSeq 2000 platform. Approximately 150 million paired-end reads were generated per sample (**Table S2**).

| **Sample ID Number** | **Sample Label** | **Tissue Source** | **Patient age (days)** | ***MYH6* Genotype** | **Gender** | **Valve Status** | **Sequencing Depth** |
| --- | --- | --- | --- | --- | --- | --- | --- |
| 1 | F3d | Right Ventricle | 3 | R443P | Female | MA/AA | 149,735,416 |
| 2 | F7d | Right Ventricle | 7 | WT | Female | MA/AA | 165,628,320 |
| 3 | M6m | Right Ventricle | 194 | D588A | Male | MS/AA | 151,824,290 |
| 4 | M7m | Right Ventricle | 221 | WT | Male | MS/AA | 172,562,906 |
| 5 | M3y | Ventricular Apex | 1,092 | K850del | Male | ND | 160,675,160 |
| 6 | F5y | Ventricular Apex | 2,121 | WT | Female | ND | 172,214,016 |
| 7 | F3d | Atrial Septum | 3 | R443P | Female | MA/AA | 228,840,886 |
| 8 | F4d | Atrial Septum | 4 | WT | Female | MA/AA | 134,240,272 |
| 9 | F4d | Atrial Septum | 4 | E1584K | Female | MS/AS | 195,114,174 |
| 10 | F4d | Atrial Septum | 4 | WT | Female | MS/AS | 113,943,776 |
| 11 | M4y | Atrial Septum | 1,649 | S385L & M436V | Male | MA/AA | 117,550,316 |
| 12 | M4y | Atrial Septum | 1,603 | WT | Male | MA/AA | 132,787,626 |
| 13 | M6d | Atrial Septum | 6 | D588A | Male | MS/AA | 148,261,692 |
| 14 | M5d | Atrial Septum | 5 | WT | Male | MS/AA | 132,187,010 |
| 15 | M8d | Atrial Septum | 8 | A964S | Male | MS/AA | 149,929,370 |
| 16 | M8d | Atrial Septum | 8 | WT | Male | MS/AA | 126,431,792 |

**Table S2. Demographic and extended information for the selected 16 RNA samples.** These samples correspond to **Figure 4a** in the manuscript. Columns 1-3 denote the Figure 4 legend and tissue source. Column 4 is patient age (days) at time of sample collection, column 5 lists the MYH6 variant, column 6 is gender, column 7 is cardiac anatomy where MA= mitral valve atresia, MS= mitral valve stenosis, AA= aortic valve atresia, AS= aortic valve stenosis, and column 8 lists sequencing depth.

*Cardiac transcriptome data analysis*

Illumina HiSeq 2000 paired-end reads were mapped to the human genome (NCBI b37) using Bowtie([11](#_ENREF_11)) under RSEM 1.2.7.([4](#_ENREF_4)) Quantification to posterior mean estimate TPM was performed by RSEM across a transcriptome reference of 38,642 RefSeq and ERCC transcripts. Transcripts were analyzed with edgeR, calculating normalization factors, then estimating generalized linear modeling (GLM) common, trended and tagwise dispersions. A likelihood ratio test evaluated each transcript’s model significance using sample pairings as covariates. Results were filtered to retain those transcripts with absolute log fold change and expression (logCPM) greater than one. Values at p<1x10^-2^ were considered statistically significant (**Table S3**). “Highly expressed genes” were defined as above.

| **Gene** | **Transcript** | **P** | **% Expression *MYH6* Variant Carriers vs. Non-Carriers** |
| --- | --- | --- | --- |
| **TNNT2** | NM_001001431 | 2.81 x 10^-3^ | 1231% |
| **MYL2** | NM_000432 | 3.94 x 10^-3^ | 366% |
| **MYH7** | NM_000257 | 7.06 x 10^-4^ | 346% |
| **ACTA1** | NM_001100 | 5.13 x 10^-3^ | 315% |
| **TPM2** | NM_003289 | 8.61 x 10^-4^ | 221% |
| ENO3 | NM_053013 | 1.90 x 10^-4^ | 312% |
| COX6A2 | NM_005205 | 6.99 x 10^-3^ | 250% |
| HHATL | NM_020707 | 2.64 x 10^-3^ | 241% |
| ALDOA | NM_184043 | 2.54 x 10^-3^ | 210% |
| EEF1B2 | NM_001037663 | 5.33 x 10^-3^ | 50% |
| HNRNPA1 | NM_002136 | 1.51 x 10^-3^ | 46% |
| RPL12 | NM_000976 | 8.58 x 10^-3^ | 45% |
| RPS3A | NM_001006 | 3.97 x 10^-3^ | 42% |
| H3F3AP4 | NR_002315 | 1.74 x 10^-3^ | 39% |
| RPS27A | NM_001177413 | 2.90 x 10^-4^ | 36% |
| EEF1A1 | NM_001402 | 3.26 x 10^-3^ | 36% |
| RPL9 | NM_000661 | 1.20 x 10^-3^ | 28% |
| RPL17 | NM_001035006 | 3.08 x 10^-4^ | 23% |
| RPSA | NM_002295 | 6.92 x 10^-4^ | 22% |
| RPL41 | NM_021104 | 1.72 x 10^-3^ | 21% |
| RPS26 | NM_001029 | 3.41 x 10^-3^ | 21% |
| RPL21 | NM_000982 | 1.16 x 10^-4^ | 15% |

**Table S3. Stage 4: Paired test between eight *MYH6*-variant carrying tissue samples and eight wildtype tissue samples in HLHS subjects: most differentially expressed genes.** Analysis with R package edgeR operating on RSEM TPM scores for 16 samples arranged in eight pairs yields a table of 38,663 gene transcripts, of which only these 22 passed filtering thresholds for significantly differential percentage of change associated with *MYH6* variant carriers. Column 1 and 2 denote gene name and transcript ID, column 3 lists P values calculated in Stage 4 using edgeR, column 4 denotes gene expression of *MYH6* variant carriers relative to non-carriers. Highlighted genes are associated with sarcomere structure and striated muscle contractility.

*MYH7 quantitative RT-PCR*

Illumina TruSeq RNA sample preparation of cDNA libraries was performed according to manufacturer’s instructions (San Diego, CA). Samples were subsequently quantified on an Agilent Bioanalyzer 2100. Real-time PCR was conducted on a Quantstudio 7 Flex instrument (Thermo Scientific, Waltham, MA) starting with 5 ng of each cDNA product, amplified in 10 microliter multiplex reactions using commercially available gene expression assays specific for the *MYH7* gene (Hs01110598-g1; FAM labelled, Thermo Scientific) and the commonly used housekeeping gene *GAPDH* (Hs99999905_m1; VIC labelled, Thermo Scientific). Quantitation of mRNA expression was determined using the delta delta Ct method and normalized to *GAPDH.*

*Western blotting & densitometry*

Lysates from ~15 mg frozen cardiac tissue (ventricle or atrium) prepared in 50 mM Tris-HCl (pH 7.4), 150 mM NaCl, 1.0% Triton X-100 and 0.1% deoxycholate containing EDTA and HALT protease inhibitors (Thermo Scientific, Waltham,MA; 87786) were sonicated, followed by determination of total protein concentration using the Bradford assay. Samples containing 2.5 µg total protein were separated under non-reducing conditions on 4-20% SDS-PAGE Mini-PROTEAN TGX gradient gels (Bio-Rad Laboratories, Hercules, CA) and transferred at 4 degrees in 15% methanol Tris-glycine buffer to Immobilon-P polyvinylidene difluoride membranes (Millipore, Danvers, MA). Membranes were blocked in buffer containing 20 mM Tris-HCl pH 7.6, 137 mM NaCl, 0.2% Tween-20 and 0.1% BSA and interrogated with a primary mouse monoclonal antibody specific for human β-myosin heavy chain (DSHB, University of Iowa; A4.951-c, 1:2000) and a rabbit polyclonal antibody raised against human GAPDH as a loading control (Abcam, Cambridge, MA; ab9485, 1:2500). Imaging was accomplished using an HRP-conjugated secondary antibody (goat anti-mouse IgG (H + L) to detect mouse antibodies, and HRP-conjugated protein A to detect rabbit antibodies, respectively (Thermo Scientific, Pierce Protein Biology Products, Waltham, MA; 31430, 32400, 1:10,000 each). The Super Signal West Pico Chemiluminescent substrate (Thermo Scientific, Waltham, MA; 34080) was used to visualize bands on a Bio-Rad ChemiDoc XRS (Hercules, CA) system.  Signal linearity was assured via titration of total loaded protein. Densitometry of bands was accomplished using Image Lab v 4.0.1 software (Bio-Rad). Quantities of β-myosin heavy chain relative to GAPDH were obtained by evaluating eight samples five times each; therefore 40 values of expression (relative intensity of β-MHC to GAPDH) are shown in **Table S4**.

| Tissue Sample Number | | | Ratio beta-MHC to GAPDH | | |
| --- | --- | --- | --- | --- | --- |
| MUT | WT | Immunoblot Gel | MUT | WT | Change |
| 3 | 4 | 1 | 301% | 269% | 112% |
| 3 | 4 | 2 | 305% | 302% | 101% |
| 3 | 4 | 3 | 174% | 127% | 138% |
| 3 | 4 | 4 | 171% | 148% | 115% |
| 3 | 4 | 5 | 114% | 98% | 116% |
| 5 | 6 | 1 | 185% | 170% | 109% |
| 5 | 6 | 2 | 229% | 157% | 146% |
| 5 | 6 | 3 | 164% | 73% | 225% |
| 5 | 6 | 4 | 111% | 96% | 116% |
| 5 | 6 | 5 | 77% | 67% | 115% |
| 7 | 8 | 1 | 196% | 150% | 131% |
| 7 | 8 | 2 | 244% | 150% | 162% |
| 7 | 8 | 3 | 167% | 122% | 137% |
| 7 | 8 | 4 | 78% | 72% | 107% |
| 7 | 8 | 5 | 55% | 45% | 121% |
| 15 | 16 | 3 | 170% | 68% | 248% |
| 15 | 16 | 4 | 109% | 36% | 300% |
| 15 | 16 | 5 | 53% | 19% | 277% |
| 15 | 16 | 6 | 404% | 64% | 635% |
| 15 | 16 | 7 | 249% | 84% | 295% |

**Table S4. Protein expression levels of *MYH7* relative to *GAPDH*, measured by Western blot.** Data corresponds to **Figure S2**, which represents one of the seven independent immunoblots listed here. Sample ID numbers match from table S2. Ratio refers to the magnitude of *MYH7* over *GAPDH* fluorescence. Data were analyzed in R by GLM/ANOVA with significance defined as p<0.01.

The analysis is performed in R with GLM /ANOVA by the following source code:

D<-read.csv("myh7_gapdh.csv",header=TRUE) #data from table S4, reformatted

D$Group <- relevel(as.factor(D$Group),2) #WT/MUT

D$Gel <- as.factor(D$Gel)

D$Person <- as.factor(D$Person)

GO2 <- glm(Ratio ~ Pairing+Gel+Group, data=D)

anova(GO2,test="Chisq")

summary(GO2)

# without factors, Ratio by Group is p=0.0231 # AIC: 102

# without pairing, just gel+group is p=0.00183 # AIC:78

# without gel, just pairing+group is p=0.01866 # AIC:101

# with pairing all pair+gel+group is p=0.000355 # AIC:66

# Adding the Group=Mutant Term helps by p=5.245*10^-5

*iPSC karyotyping*

Karyotyping analysis was performed at cell passages 12-21. iPSCs cultured in mTeSR1 medium on matrigel-coated T25 flasks were sent to Wisconsin Diagnostics (Milwaukee, WI) for cytogenetic analysis using G-banded karyotyping. The cells were analyzed for chromosome integrity.

*iPSC* 🡪 *Cardiomyocyte differentiation*

All experiments were performed on cells during passages 10-40. Approximately one day before inducing cardiomyogenesis, the cells were re-coated with matrigel to create a sandwich. Upon attaining confluency, differentiation was induced (on Day 0) by moving the cultures to a normoxic environment and exchanging mTeSR1 medium for insulin-free RPMI/B27 supplemented with 9 μM CHIR99021 (Stemgent, Lexington, MA; 04-0004-2) and 10 ng/ml Activin-A (R&D Systems, Minneapolis, MN; 338-AC-005). After 20 hours (Day +1) the medium was replaced with insulin-free RPMI/B27 medium without CHIR and Activin-A. On Day +3 the medium was exchanged for insulin-free RPMI/B27 with 5 μM IWP (Tocris, Bristol, UK; 3533). Two days later (Day +5) the medium was replaced with insulin-free RPMI/B27 minus IWP. At Day +7, the medium was changed to 2.0 ml RPMI/B27 with insulin, followed by identical medium changes at 2 day intervals thereafter. Experiments were terminated at Day +9 or +10 as indicated, after which duplicate cultures were evaluated for percentages of cardiac troponin-T-positive (cTnT+) cells via flow cytometry, which was correlated with the extent of myosin heavy chain (MF-20) immunostaining performed on parallel cultures.

*iPSC analysis - immunostaining & flow cytometry & qPCR preparation*

Primary antibodies employed for immunofluorescent staining were anti-myosin heavy chain monoclonal (DSHB, University of Iowa; MF20), anti-Oct4 monoclonal (Millipore, Billerica, MA; MAB-4305), or anti-Oct4 rabbit polyclonal (Santa Cruz, Dallas, TX; sc-9081), anti-sarcomeric α-actinin monoclonal (Abcam, Cambridge, MA; ab9465) and anti-Sox17 goat polyclonal (R&D Systems, Minneapolis, MN; AF1924). Evaluation of sarcomere organization (Fig. S4) was performed on Day 60+ of CM (cardiomyocyte) differentiation. To quantify cells exhibiting normal vs. defective sarcomere organization, mass-cultured CMs on differentiation day 65 were sub-cultured onto matrigel at a density of 10^4^ cells / cm^2^ until differentiation day 68, when they were co-immunostained with MF20 (AF488) to inform myocyte identity, and with sarcomere α-actinin (AF594) to image sarcomere organization. CMs judged to contain normal sarcomere organization exhibited crisp α-actinin staining with elongated sarcomeric ladders containing wide Z-bands. In CMs judged to contain defective sarcomere organization, most of the cellular area exhibited blurred α-actinin staining wherein sarcomeric ladders displayed truncated to punctate α-actinin deposits.

Antibodies for flow cytometry were monoclonal anti-cardiac Cardiac Troponin T (TNNT2; Thermo Scientific, Waltham, MA; MS-295-R7) and goat anti-mouse IgG1 secondary antibody conjugated with Alexa Fluor 488 (Invitrogen, New York; A21121).

For the qPCR determination, the cells in each culture were harvested by scraping on the indicated days and RNA was purified (see above cardiac transcriptome sequencing). cDNA was synthesized using Quanti-Tect Reverse Transcription according to the manufacturer’s (Qiagen, Valencia, CA). Identical quantities of RNA from each sample were reverse-transcribed (250-1,000 ng, depending on the experiment). For real-time PCR, 10 ng of each cDNA was used as template by suspending in Taqman Master Mix (Thermo Scientific, Waltham,MA) using conditions described above (see *MYH7 quantitative RT-PCR)* followed by amplification using a Quanti Studio 7 Flex (Thermo Scientific, Waltham, MA).


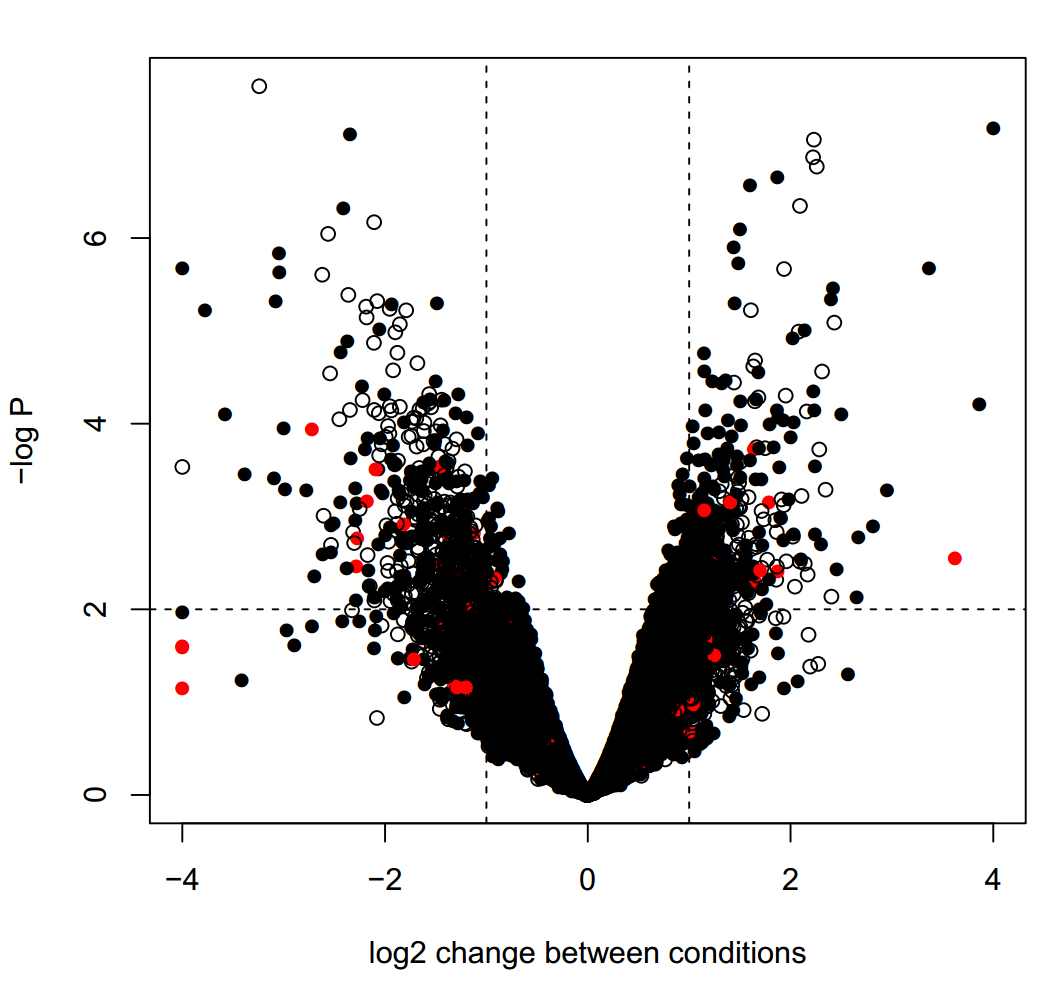


**Figure S1**. Volcano plot of differential gene expression (**Stage 4**).

To illustrate a common RNA-Seq differential expression analysis, we create a scatterplot of genes’ change and significance. In a volcano plot, the x axis is the log fold change between conditions, such that most genes are in the center at logFC=0, and genes highly upregulated by the test condition (here *MYH6* carrier status) are at the far right. This plot has been truncated to the range (-4,4) for visibility. The vertical axis is the negative log10 of the p value of the statistical test for difference between groups. Therefore a 2 corresponds to p=1 x 10^-2^, and a 4 corresponds to p=1 x 10^-4^. Dashed lines are drawn at our thresholds for interesting results, at abs(logFC)=1 and p=1 x 10^-2^. Of 38,663 points on this diagram, 975 are found in the upper left and right quadrants, indicating nominal significance. In this figure, points are empty circles when the overall mean expression among the test subjects was low (log2CPM < 1) and filled circles when moderate-to-high (log2CPM>1). To restrict our result to possibly important cardiac development genes, we also color the genes red when the average expression among all samples was greater than the 99^th^ percentile. The “High expression” filter reduces the 975 results to 24. Two of the 24 are exogenous spike-in control sequences and are omitted from Table S3, such that Stage 4 results in 22 differentially “highly expressed genes” among *MYH6* carriers vs non-carriers.


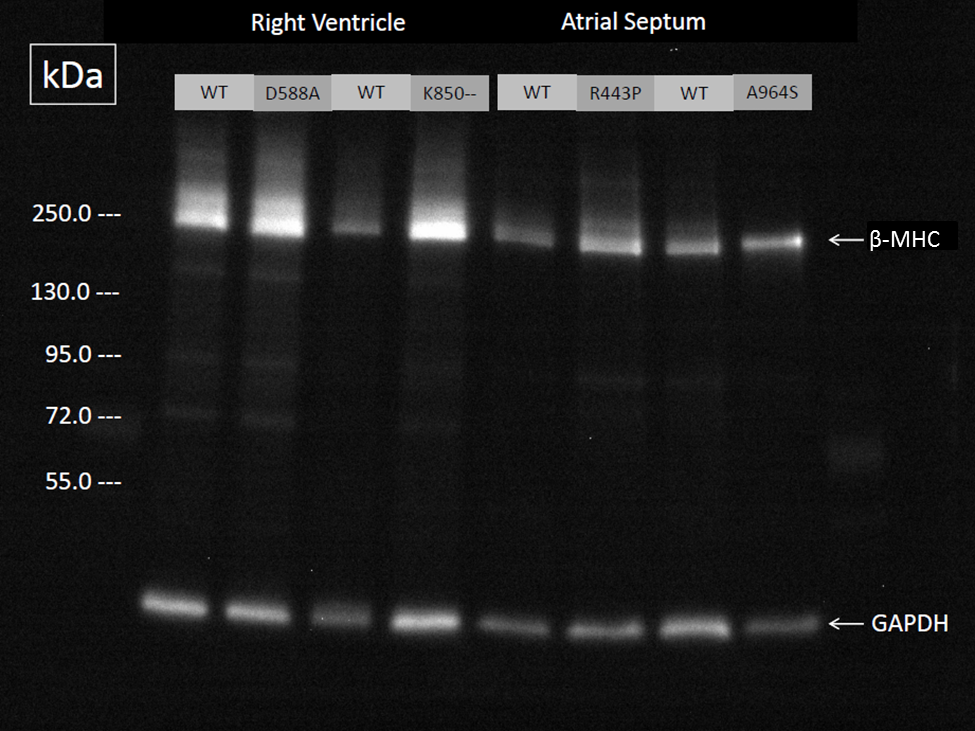


**Figure S2.** **Western blot analysis**. Representative western blot showing levels of β-myosin heavy chain protein relative to GAPDH in subjects with *MYH6* variants. This presents the entire immunoblot in order to demonstrate antibody specificity; strips are shown in **Figure 4b top panel**.


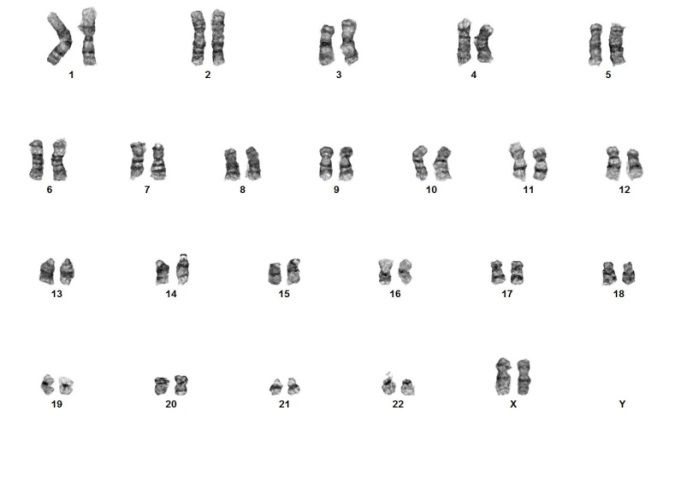

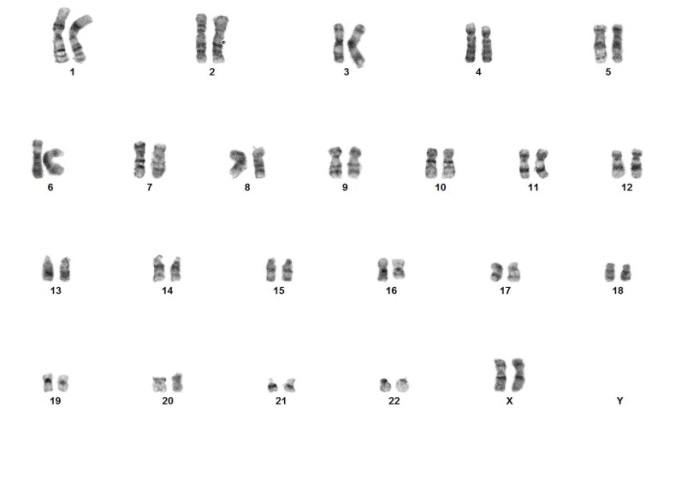


**F MYH6:R443P Proband**

**F MYH6:R443P unaffected parent**


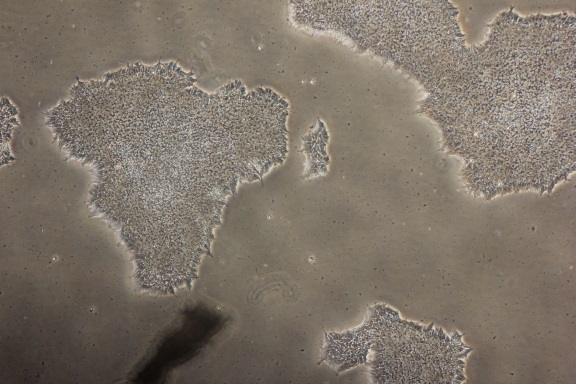

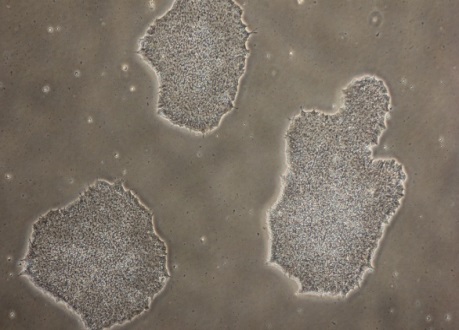


**Figure S3. Karyotype and colony morphology of iPSCs derived from the HLHS proband and unaffected parent of family *MYH6*-R443P.**

iPSCs-derived from the HLHS proband (individual IV.3) and unaffected parent (individual II.2) of the family depicted in Figure 2 exhibited no abnormalities in chromosomal number or banding pattern (upper panels). The pluripotent cell colonies (lower panels) have similar morphology to human embryonic stem cells.

**
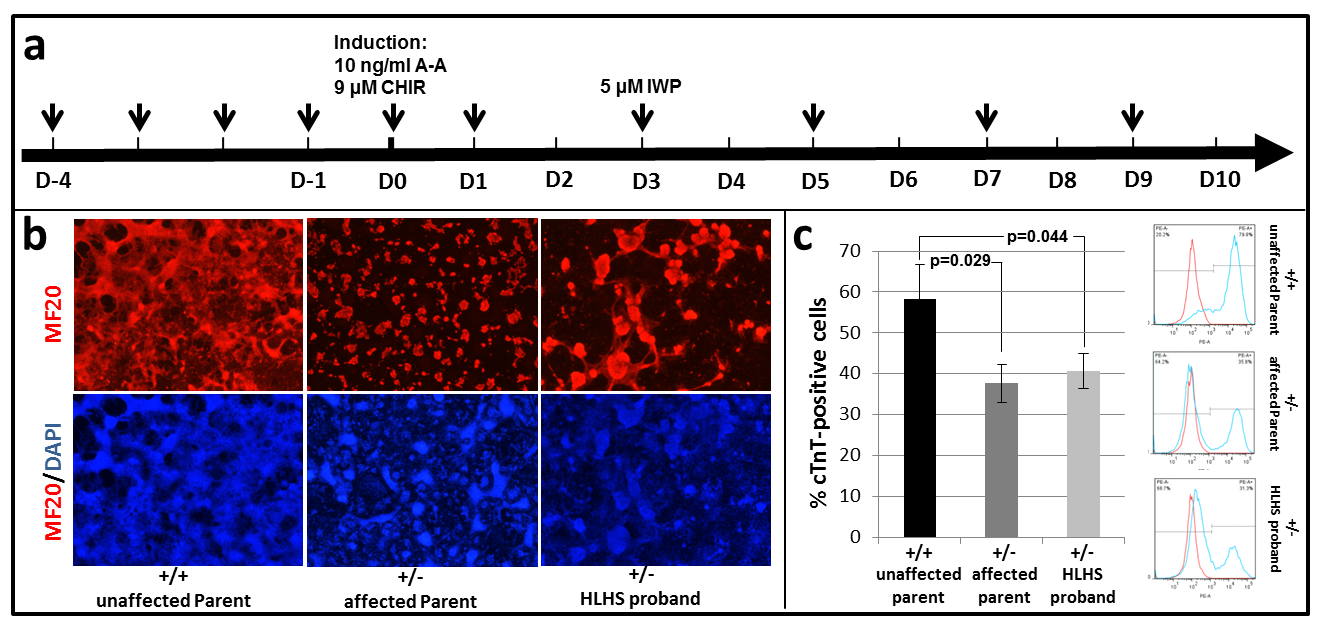
**

**Figure S4. iPSC-derived cardiomyocytes from the HLHS proband and affected parent of family *MYH6*-D588A exhibit defective cardiomyogenesis.**

**Panel a**, scheme for differentiating cardiomyocytes from iPSCs. **Panel b**, immunostaining of α-myosin heavy chain differentiation at Day 10 showing defective cardiomyogenesis in iPSCs derived from the HLHS proband and carrier father (affected parent). **Panel c**, flow cytometry of cells cultured in parallel with those in panel b, revealing decreased percentages of cTnT-positive cells at Day 10. Data in panel c were compiled from three independent experiments, each of which interrogated three iPSC lines derived from each individual. The p-values were calculated using Student’s t-test (two-tailed, equal variance); vertical lines = ±SEM.

**References:**

1. **Adzhubei IA, Schmidt S, Peshkin L, Ramensky VE, Gerasimova A, Bork P, Kondrashov AS, and Sunyaev SR**. A method and server for predicting damaging missense mutations. *Nature methods* 7: 248-249, 2010.

2. **Cooper GM, Stone EA, Asimenos G, Green ED, Batzoglou S, and Sidow A**. Distribution and intensity of constraint in mammalian genomic sequence. *Genome research* 15: 901-913, 2005.

3. **DePristo MA, Banks E, Poplin R, Garimella KV, Maguire JR, Hartl C, Philippakis AA, del Angel G, Rivas MA, Hanna M, McKenna A, Fennell TJ, Kernytsky AM, Sivachenko AY, Cibulskis K, Gabriel SB, Altshuler D, and Daly MJ**. A framework for variation discovery and genotyping using next-generation DNA sequencing data. *Nat Genet* 43: 491-498, 2011.

4. **Li B, and Dewey CN**. RSEM: accurate transcript quantification from RNA-Seq data with or without a reference genome. *BMC bioinformatics* 12: 323, 2011.

5. **Li H, and Durbin R**. Fast and accurate short read alignment with Burrows-Wheeler transform. *Bioinformatics* 25: 1754-1760, 2009.

6. **McKenna A, Hanna M, Banks E, Sivachenko A, Cibulskis K, Kernytsky A, Garimella K, Altshuler D, Gabriel S, Daly M, and DePristo MA**. The Genome Analysis Toolkit: a MapReduce framework for analyzing next-generation DNA sequencing data. *Genome research* 20: 1297-1303, 2010.

7. **McLaren W, Pritchard B, Rios D, Chen Y, Flicek P, and Cunningham F**. Deriving the consequences of genomic variants with the Ensembl API and SNP Effect Predictor. *Bioinformatics* 26: 2069-2070, 2010.

8. **Ng PC, and Henikoff S**. SIFT: Predicting amino acid changes that affect protein function. *Nucleic acids research* 31: 3812-3814, 2003.

9. **Paila U, Chapman BA, Kirchner R, and Quinlan AR**. GEMINI: integrative exploration of genetic variation and genome annotations. *PLoS computational biology* 9: e1003153, 2013.

10. **Tomita-Mitchell A, Mahnke DK, Struble CA, Tuffnell ME, Stamm KD, Hidestrand M, Harris SE, Goetsch MA, Simpson PM, Bick DP, Broeckel U, Pelech AN, Tweddell JS, and Mitchell ME**. Human gene copy number spectra analysis in congenital heart malformations. *Physiol Genomics* 44: 518-541, 2012.

11. **Trapnell C, Pachter L, and Salzberg SL**. TopHat: discovering splice junctions with RNA-Seq. *Bioinformatics* 25: 1105-1111, 2009.

12. **Wain LV, Verwoert GC, O’Reilly PF, Shi G, Johnson T, Johnson AD, Bochud M, Rice KM, Henneman P, Smith AV, Ehret GB, Amin N, Larson MG, Mooser V, Hadley D, Dörr M, Bis JC, Aspelund T, Esko T, Janssens ACJW, Zhao JH, Heath S, Laan M, Fu J, Pistis G, Luan Ja, Arora P, Lucas G, Pirastu N, Pichler I, Jackson AU, Webster RJ, Zhang F, Peden JF, Schmidt H, Tanaka T, Campbell H, Igl W, Milaneschi Y, Hotteng J-J, Vitart V, Chasman DI, Trompet S, Bragg-Gresham JL, Alizadeh BZ, Chambers JC, Guo X, Lehtimäki T, Kühnel B, Lopez LM, Polašek O, Boban M, Nelson CP, Morrison AC, Pihur V, Ganesh SK, Hofman A, Kundu S, Mattace-Raso FUS, Rivadeneira F, Sijbrands EJG, Uitterlinden AG, Hwang S-J, Vasan RS, Wang TJ, Bergmann S, Vollenweider P, Waeber G, Laitinen J, Pouta A, Zitting P, McArdle WL, Kroemer HK, Völker U, Völzke H, Glazer NL, Taylor KD, Harris TB, Alavere H, Haller T, Keis A, Tammesoo M-L, Aulchenko Y, Barroso I, Khaw K-T, Galan P, Hercberg S, Lathrop M, Eyheramendy S, Org E, Sõber S, Lu X, Nolte IM, Penninx BW, Corre T, Masciullo C, Sala C, Groop L, Voight BF, Melander O, O’Donnell CJ, Salomaa V, d’Adamo AP, Fabretto A, Faletra F, Ulivi S, Del Greco MF, Facheris M, Collins FS, Bergman RN, Beilby JP, Hung J, Musk AW, Mangino M, Shin S-Y, Soranzo N, Watkins H, Goel A, Hamsten A, Gider P, Loitfelder M, Zeginigg M, Hernandez D, Najjar SS, Navarro P, Wild SH, Corsi AM, Singleton A, de Geus EJC, Willemsen G, Parker AN, Rose LM, Buckley B, Stott D, Orru M, Uda M, LifeLines Cohort S, van der Klauw MM, Zhang W, Li X, Scott J, Chen Y-DI, Burke GL, Kähönen M, Viikari J, Döring A, Meitinger T, Davies G, Starr JM, Emilsson V, Plump A, Lindeman JH, ’t Hoen PAC, König IR, EchoGen c, Felix JF, Clarke R, Hopewell JC, Ongen H, Breteler M, Debette S, DeStefano AL, Fornage M, AortaGen C, Mitchell GF, Group CCHFW, Smith NL, KidneyGen c, Holm H, Stefansson K, Thorleifsson G, Thorsteinsdottir U, consortium CK, Cardiogenics c, CardioGram, Samani NJ, Preuss M, Rudan I, Hayward C, Deary IJ, Wichmann HE, Raitakari OT, Palmas W, Kooner JS, Stolk RP, Jukema JW, Wright AF, Boomsma DI, Bandinelli S, Gyllensten UB, Wilson JF, Ferrucci L, Schmidt R, Farrall M, Spector TD, Palmer LJ, Tuomilehto J, Pfeufer A, Gasparini P, Siscovick D, Altshuler D, Loos RJF, Toniolo D, Snieder H, Gieger C, Meneton P, Wareham NJ, Oostra BA, Metspalu A, Launer L, Rettig R, Strachan DP, Beckmann JS, Witteman JCM, Erdmann J, van Dijk KW, Boerwinkle E, Boehnke M, Ridker PM, Jarvelin M-R, Chakravarti A, Abecasis GR, Gudnason V, Newton-Cheh C, Levy D, Munroe PB, Psaty BM, Caulfield MJ, Rao DC, Tobin MD, Elliott P, and van Duijn CM**. Genome-wide association study identifies six new loci influencing pulse pressure and mean arterial pressure. *Nature genetics* 43: 1005-1011, 2011.
